# Unravelling the Novel Effects of Three Volatile Compounds in Preventing Fibril Formation of Disease Related Tau and α-Synuclein Proteins- Towards Identifying Candidate Aromatic Substances for Treating Neurodegenerative Diseases

**DOI:** 10.3389/fphar.2022.793727

**Published:** 2022-03-22

**Authors:** Zahra Moeini, Zahra Seraj, Toktam Zohoorian Abootorabi, Mohammadreza Ashrafi-Kooshk, Gholamhossein Riazi, Ali Akbar Saboury, Arefeh Seyedarabi

**Affiliations:** Department of Biochemistry, Institute of Biochemistry and Biophysics, University of Tehran, Tehran, Iran

**Keywords:** Volatile compounds, Cinnamaldehyde, Phenyl ethyl alcohol, TEMED, Tau protein, α-synuclein, Fibrillation, Amyloid formation

## Abstract

**Background:** The aggregation of tau and α-synuclein into fibrillary assemblies in nerve cells is the molecular hallmark of Alzheimer’s and Parkinson’s diseases, respectively. In our previous studies, we investigated the anti-amyloidogenic effects of three different aroma-producing (volatile) compounds including cinnamaldehyde, phenyl ethyl alcohol, and TEMED on the fibrillation process of HEWL, as a model protein. Our previous results showed that while TEMED was able to completely stop the process of fibril formation, cinnamaldehyde and phenyl ethyl alcohol gave rise to oligomeric/protofibrillar forms and were involved in the entrapment of intermediate species of HEWL. In this study, we investigated the anti-amyloidogenic effect of the same three volatile compounds on recombinantly produced tau and α-synuclein proteins.

**Methods:** The thioflavin T fluorescence assay, circular dichroism, SDS-PAGE/native-PAGE, dynamic light scattering, and atomic force microscopy were used, where necessary, to further our understanding of the inhibitory effects of the three volatile compounds on the fibril formation of tau and α-synuclein proteins and allow for a comparison with previous data obtained for HEWL.

**Results:** Our results revealed that contrary to the results obtained for HEWL (a globular protein), the volatile compound TEMED was no longer able to prevent fibril formation in either of the natively unstructured tau or α-synuclein proteins, and instead, cinnamaldehye and phenyl ethyl alcohol, in particular, had the role of preventing fibril formation of tau or α-synuclein.

**Conclusion:** The results of this study further emphasized the exclusion of HEWL as a model protein for fibrillation studies and highlighted the importance of studying brain-related proteins such as tau or α-synuclein and the need to assess the effects of volatile compounds such as cinnamaldehye and phenyl ethyl alcohol as potential substances in the treatment of neurodegenerative diseases.

## 1 Introduction

Research on neurodegenerative diseases has shown that a common pathogenic factor in many of the diseases is characterized by a specific protein or peptide that aggregates (Trzeœniewska et al., 2004; Jangholi et al., 2016). Diseases such as Alzheimer’s (AD), Parkinson’s (PD), prion, and Huntington’s (HD) occur as a result of protein aggregation (Shastry, 2003). As such, intracellular accumulation of microtubule-associated tau protein in the form of filamentous aggregates is an obvious pathology of neurodegenerative diseases, including AD (Sahara et al., 2008), while α-synuclein aggregates have been associated with PD (Shastry, 2003).

Additionally, based on scientific reports, it was shown that the first inclusions of Lewy bodies and α-synuclein aggregates in PD were present in the olfactory bulb (Goedert, 2015), while a decreased sense of smell was reported in the early stages of AD (Eriksson et al., 1998; Perez et al., 2001). With regard to the decreased or loss of sense of smell, also known as anosmia, viral infections such as coronavirus disease 2019 (COVID-19) (Han et al., 2020) have also been reported to cause olfactory dysfunction; recent studies have shown that about 20–85% of the COVID-19 patients had olfactory dysfunction or anosmia (Giacomelli et al., 2020; Vaira et al., 2020). Furthermore, growing evidence suggests that COVID-19 infection is often not limited to the nasal cavity and upper respiratory tract but also enters the central nervous system under unclear circumstances, leading to the onset of neuronal invasion and the potential for neurodegenerative risk (Han et al., 2020). Moreover, it should be mentioned that anosmia also occurs in epilepsy, migraine, meningitis, and CNS disorders (Hong et al., 2012). As it is clear, anosmia is a common symptom shared between neurodegenerative diseases and COVID-19 infection as well as epilepsy, migraine, meningitis, and CNS disorders. There is evidence to suggest the presence of positive effects of aroma in controlling the central nervous system (Koo et al., 2003; Koo et al., 2004), as well as the fact that stimulating the sense of smell can improve patients’ cognitive function and allow nerve regrowth through odour-producing molecules (Jimbo et al., 2009). Therefore, the importance of aromatherapy can be highlighted here whereby aroma-producing/volatile small compounds can be used as therapeutic molecules to prevent protein aggregation or fibril formation and potentially restore symptoms such as anosmia. However, there has not been any research as such to reveal the therapeutic effects of aroma and aromatherapy by molecular studies (Lee et al., 2012). As a result, we were initially encouraged to investigate the effect of volatile form of three different compounds including cinnamaldehyde and phenyl ethyl alcohol, which give the pleasant smell of cinnamon and rose flower, respectively, and also N,N,N′,N′-tetramethylethylenediamine, an unpleasant smelling chemical compound similar to putrescine and cadaverine, on hen egg white lysozyme (HEWL) fibrillation, as a model protein in fibrillation studies (Seraj et al., 2018). However, we concluded that HEWL was not the correct model protein (Seraj et al., 2019; Seraj et al., 2020) and decided that fibrillation studies should be performed on disease-related proteins. Therefore, in this study, we evaluated the effect of the same three different volatile compounds on recombinant tau and α-synuclein protein fibrillation, instead of HEWL. Tau and α-synuclein protein over-production and purification were achieved using bacterial cell culture followed by nickel affinity chromatography. The thioflavin T (ThT) fluorescence assay, circular dichroism (CD), SDS-PAGE/native-PAGE, atomic force microscopy (AFM), and dynamic light scattering (DLS) were used, where necessary, to further our understanding of the inhibitory effects of the three volatile compounds on the fibril formation of tau and α-synuclein proteins and allow for a comparison with our previous data obtained on HEWL.

## 2 Materials and Methods

### 2.1 Materials

Resin of Ni-NTA gravity-flow agarose for efficient purification of His-tagged tau protein was obtained from QIAGEN. Thioflavin T (ThT), N,N,N′,N′-tetramethylethylenediamine (TEMED; CAS number 110–189), trans-cinnamaldehyde (Cin; Lot number MKBV8774V), and phenyl ethyl alcohol (PEA; catalog number W285803) were all purchased from Sigma-Aldrich. Heparin (average molecular mass of 15,000 Da) was purchased from Celsus Laboratories (Cincinnati, OH). The tau34-pET21 construct, which encoded the 412 amino acid tau protein, was kindly gifted by Dr. MA. Nasiri (University of Tehran, Iran). The protein marker was purchased from SMOBiO. All other chemicals were of analytical grade. All solutions were prepared using double-distilled water.

### 2.2 Methods

#### 2.2.1 Recombinant Tau Protein Over-Production and Purification

The His-tagged 1N4R tau cDNA (GenBank NO: P10636) was inserted into the pET-21a (+) plasmid vector using NdeI/XhoI and transformed into *E. coli* BL21 (DE3) for expression (Khalili et al., 2014). Briefly, *E. coli* was grown at 37°C in 25 ml of LB and 100 μg/ml ampicillin and cultured for 14 h before being transferred into a 1 L LB culture. After induction with 0.5 mM IPTG at 37°C for 4 h, the harvested cells were resuspended in lysis buffer (50 mM Tris-HCl of pH 7.6, 250 mM NaCl, 30 mM imidazole, 5 mM DTT, and 1 mM PMSF) and then sonicated on ice. Cell debris was precipitated by centrifugation at 20,000 g for 20 min at 4°C, after which the supernatant was filtered and then added to a volume of 1 ml Ni-NTA agarose resin. Tau protein was eluted from the beads by affinity precipitation in elution buffer containing 50 mM Tris buffer (pH 7.6), 250 mM NaCl, and 100 mM imidazole (Hilbrig and Freitag, 2003). The protein was then stored at –70°C. To assess tau protein over-production and purity, 10 µL of tau protein solution from each step of over-production and purification was loaded on a 12% SDS polyacrylamide gel. Staining was performed with Coomassie Brilliant Blue R-250 stain. Protein concentration was determined by UV absorbance at 280 nm. The extinction coefficient of 1N/4R tau (7,450) based on the amino acid sequence was obtained from the ExPASy server based on the amino acid sequence of the tau protein.

#### 2.2.2 Recombinant α-Synuclein Protein Over-Production and Purification

The expression of human wild-type α-synuclein was performed in *E. coli* BL21 (DE3) using a pT7-7–based expression system (Dehghani et al., 2020). After induction with 1 mM IPTG for 4 h at 37°C, cell pellets were harvested by centrifugation at 7,000 rpm at 4°C for 20 min. The pellet was resuspended in 20 mM Tris-HCl of pH 8.0, 1 mM EDTA, and 1 mM PMSF. Cell lysis was carried out by sonication followed by boiling for 20 min. The resultant suspension was centrifuged at 14,000 rpm for 20 min at 4°C. The supernatant was used for the next step involving an ammonium sulfate precipitation step to selectively precipitate the α-synuclein protein. The solution was stirred for 1 h at 4°C and centrifuged at 14,000 rpm for 20 min at 4°C. Subsequently, the pellet was dissolved in 20 mM Tris-HCl of pH 8.0, 1 mM EDTA, and 1 mM PMSF, sterile-filtered, and loaded onto a 5-ml HiTrap Q FF anion exchange chromatography column (GE Healthcare). Fractions were collected during elution at 300 mM NaCl, with a salt gradient from 0 to 600 mM NaCl, and analyzed by SDS-PAGE followed by Coomassie staining. Subsequently, fractions containing α-synuclein were dialyzed against phosphate-buffered saline (PBS) of pH 7.4 (made up of 137 mM NaCl, 2.7 mM KCl, 10 mM Na_2_HPO_4_, and 1.76 mM KH_2_PO_4_). Protein concentrations were determined by measuring the absorbance at 275 nm using an extinction coefficient of 5,600 M^−1^ cm^−1^ (Hoyer et al., 2002). Purified protein was stored at −75°C in 1 ml aliquots.

#### 2.2.3 Tau and α-Synuclein Sample Preparation and Incubation Studies

**FIGURE 1:**
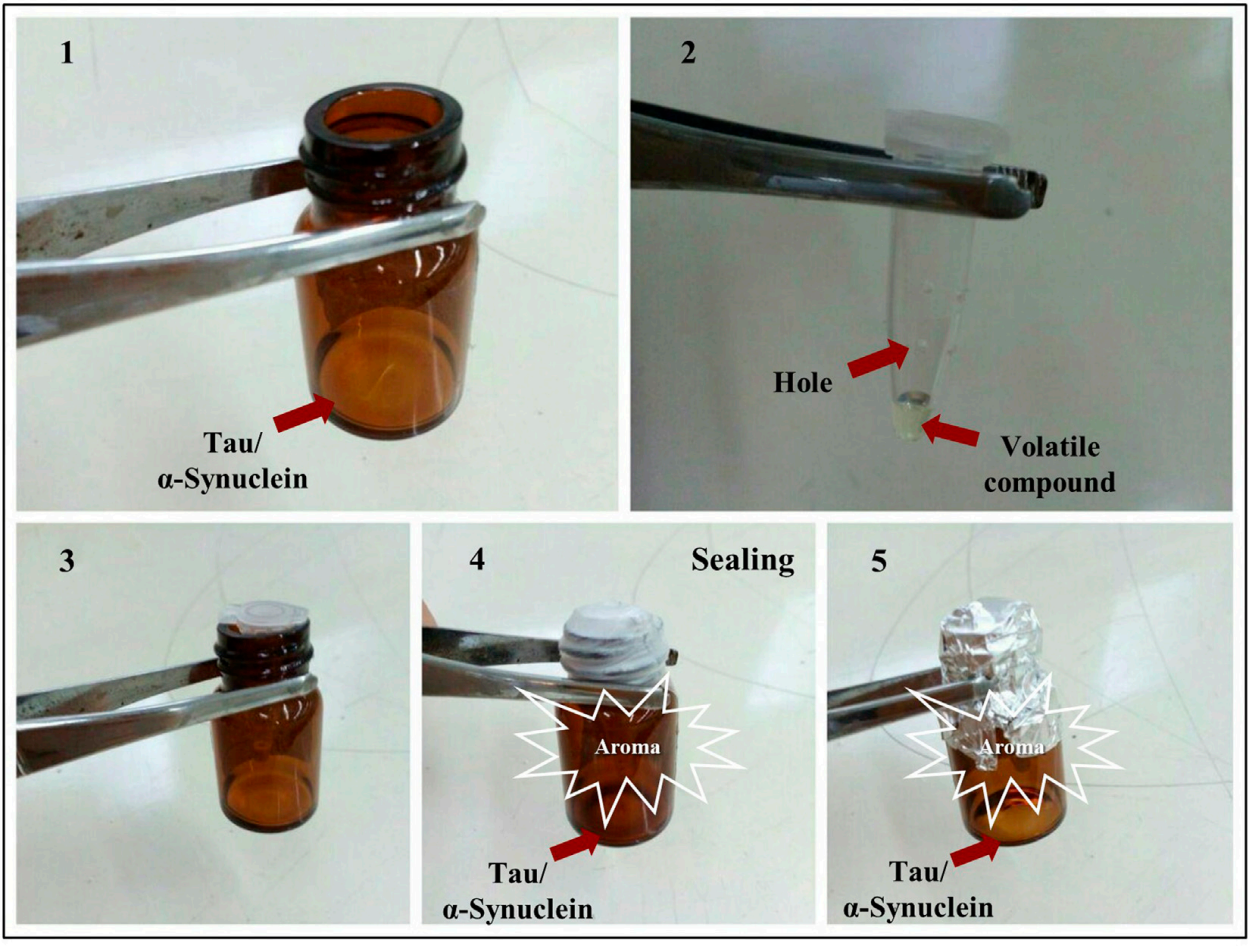
Experimental setup. (1) Tau protein sample solutions were prepared by initially adding 700 µl of a solution containing 20 µM tau protein in 50 mM Tris-HCl of pH 7.6, and 100 mM NaCl in the presence of 5 µM of Heparin and 5 mM DTT. As for α-synuclein, the sample solution contained 1 ml of 142 µM of α-synuclein in PBS of pH 7.4. Both sample solutions were placed in 20 ml bottles. (2) Holes were made in 0.5 ml Eppendorf tubes, and then 20 μl of PEA, Cin, or TEMED was added to the Eppendorf tubes. (3) The Eppendorf tubes with holes were placed inside the bottles. (4,5) The Eppendorf tubes placed inside the 20 ml bottles were sealed together and incubated at 37°C for 96 h for tau protein in a shaker at 500 rpm, and at 37°C for 72 h in a shaker at 220 rpm for α-synuclein, in order for the process of fibrillation to take place, in the presence of PEA, Cin, or TEMED.

The experimental setup was such that ∼460 µl of purified 30 µM stock of tau protein was added to 240 µl of 50 mM Tris-HCl of pH 7.6, 5 µM heparin, and 100 mM NaCl in the presence of 5 mM DTT (the final concentration of the tau protein was 20 µM). Subsequently, the mixture was added to the bottom of a 20 ml glass bottle. As for the sample solutions of α-synuclein, they were prepared at 142 µM in PBS of pH 7.4 and then also placed in 20 ml glass bottles. Subsequently, 20 μl volume of volatile/aroma-producing compounds Cin, PEA, and TEMED was transferred into 1.5 ml Eppendorf tubes with small holes (average diameter size of 0.15 mm) and placed inside the 20 ml bottles containing either tau or α-synuclein protein; the lids of the Eppendorf tubes and the bottles were sealed together. The holes in the Eppendorf tubes allowed diffusion of the volatile compounds into the bottles containing either tau or α-synuclein protein (Figure 1). The bottles containing the protein, as well as the Eppendorf tubes with the volatile compounds inside, were incubated at 37°C for 96 h in a shaker at 500 rpm for tau protein and at 37°C for 72 h in a shaker at 220 rpm for α-synuclein, in order for the process of fibrillation to take place.

Two controls were used in this study including “not-heated” and “not-treated” samples. For α-synuclein, the “not-heated” sample contained α-synuclein at 142 µM in PBS of pH 7.4, and for tau protein, it contained tau protein at 20 μM, 50 mM Tris-HCl of pH 7.6, and 100 mM NaCl. The “not-heated” controls were not incubated under fibrillation conditions. The “not-treated” sample for tau protein contained tau at 20 μM, 50 mM Tris-HCl of pH 7.6, and 100 mM NaCl in the presence of 5μM of Heparin and 5 mM DTT, incubated at 37°C for 96 h in a shaker at 500 rpm, while for α-synuclein, the protein was incubated at 37°C for 72 h in a shaker at 220 rpm. The “not-treated” controls were in the absence of the volatile compounds. All of the experiments including ThT, CD, SDS-PAGE/native-PAGE, AFM, and DLS were carried out using samples prepared and incubated from this stage.

#### 2.2.4 Tau Protein Kinetics Studies

Heparin has been traditionally used to initiate tau protein aggregation *in vitro*. Aggregation studies of tau protein were prepared containing 20 µM tau, 50 mM Tris-HCl of pH 7.6, and 100 mM NaCl in the presence of 5 µM of Heparin and 5 mM DTT and incubated at 37°C for 96 h in a shaker at 500 rpm. At different indicated time points of aggregation, the samples were taken and analyzed by ThT fluorescence and SDS-PAGE techniques.

#### 2.2.5 SDS-PAGE and Native-PAGE Analyses

SDS-PAGE analysis using 12% gels was used to analyze tau protein, while SDS-PAGE and native-PAGE techniques were used to assess α-synuclein using 18% gels. A pre-stained protein marker was used.

#### 2.2.6 Thioflavin T (ThT) Fluorescence Assay

The ThT fluorescence assay was used to investigate whether tau or α-synuclein was converted to amyloid-like fibrils. This experiment was performed in the presence of the polyanionic inducer heparin and reducing agent DTT for tau protein. For the ThT assay, a final concentration of 2.5 µM tau protein or 2.84 μM of α-synuclein and 25 µM ThT was used. Fluorescence intensities were recorded at 484 nm after excitation at 440 nm. Excitation and emission slit widths for tau protein were both set at 10 nm and for α-synuclein at 5 nm. Samples of “not-heated” tau protein and α-synuclein were also assessed, and their ThT emission values were recorded as controls for each experiment. The results were repeated, and a standard deviation bar was calculated for the graphs using multiple data. All fluorescence measurements were carried out using a fluorescence spectrophotometer at room temperature.

#### 2.2.7 Data Analysis of ThT Results

Plots of blank-subtracted fluorescence values versus time were analyzed with a procedure of best fit, using the following sigmoidal function:

$$F=\frac{Fmax}{1+exp\left\{-K_{app}\left(t-t_{m}\right)\right\}}$$, (1)

where F and F_max_ are the fluorescence intensities at time t and final time, respectively; t_m_ is the time taken to form half of the aggregates; and k_app_ presents the apparent rate constant of aggregation growth. F_max_ and t were used as floating parameters in the procedure of best fit (Furukawa et al., 2011).

#### 2.2.8 Circular Dichroism Spectroscopy

Circular dichroism (CD) spectra of tau protein samples were recorded within a wavelength range of 195–250 nm using an AVIV 215 spectrophotometer (Aviv Associates, Lakewood, NJ, United States). The sample preparations were the same as described before in our previous study (Seraj et al., 2018). Three scans of each sample were measured and averaged. The control buffer scans were run and then subtracted from the sample spectra. The results were plotted as ellipticity (deg. cm^2^ dmol^−1^) versus wavelength (nm).

#### 2.2.9 Atomic Force Microscopy Imaging

The structure and distributed population of tau/α-synuclein granular- or fibril-shaped aggregates were observed by quantitative atomic force microscopy (AFM) at Sharif University, Tehran, Iran. All samples, either treated or not treated with the three volatile compounds in this study, as well as the not-heated protein samples, were prepared by aliquoting 5 μl of the samples onto freshly cleaved mica followed by fixation through incubation for 30 min at room temperature. The mica surface was then washed with 100 µl deionized water and left to dry at room temperature.

#### 2.2.10 Dynamic Light Scattering

Dynamic light scattering measurements were performed using Malvern Zeta Sizer Nano ZS on α-synuclein samples. The apparatus and parameters used were the same as those described in our previous study (Seraj et al., 2018).

## 3 Results and Discussion

Previously, we reported the effect of the volatile compounds Cin, PEA, and TEMED on the process of fibril formation of HEWL, as a model protein (Seraj et al., 2018). Additionally, the cinnamon extract and Cin have previously been reported to show their effects in preventing tau protein fibril formation in solution (Peterson et al., 2009). In this study, we decided to evaluate the effect of the volatile compounds Cin, PEA, and TEMED on the fibrillation of recombinantly produced disease-related tau and α-synuclein proteins and compare with data obtained on HEWL as a model protein.

The fibrillation of tau and α-synuclein after 96 and 72 h, respectively, in the presence and absence of the volatile compounds was monitored, where necessary, by ThT fluorescence, CD, SDS-PAGE/native-PAGE, AFM, and DLS techniques, under conditions described in the methods section.

### 3.1 Tau Protein Studies

#### 3.1.1 Tau Protein Over-Production and Purification

The human central nervous system expresses six tau isoforms that range in size from 352 to 441 amino acids. Human adult tau protein has approximately equal representations of 3 and 4R tau isoforms, with 1N3R and 1N4R being the most abundant forms. The 1N4R isoform was selected in this study since investigation of the full-length adult tau protein leads to a better understanding of the nature of tau fibril formation mechanism (Robert and Mathuranath, 2007). The His-tagged 1N4R tau construct was used for expression of tau protein in bacterial cells (Khalili et al., 2014). It was reported that the assembly properties of the recombinant tau were not affected by the presence of the His-tag (Karikari et al., 2017). Tau protein was purified using the affinity chromatography method and characterized by SDS-PAGE (Karakani et al., 2014). Therefore, the purity of the expressed tau protein was confirmed using SDS-PAGE. As shown in Supplementary Figure S1, tau protein was observed as the major protein over-produced. The pre- and post-induced samples were compared in lanes 1 and 2, which showed the presence of a considerable concentrated band close to the 60 kDa marker band over-produced upon induction. Despite the fact that tau protein has an actual molecular weight of 43 kDa, it migrated close to the 60 KDa protein marker band, due to the slow migration of this protein, which was also previously observed in other studies (Goedert and Jakes, 1990).

#### 3.1.2 Kinetic Studies of Tau Protein Fibril Formation Using Heparin in the Presence of DTT

**FIGURE 2:**
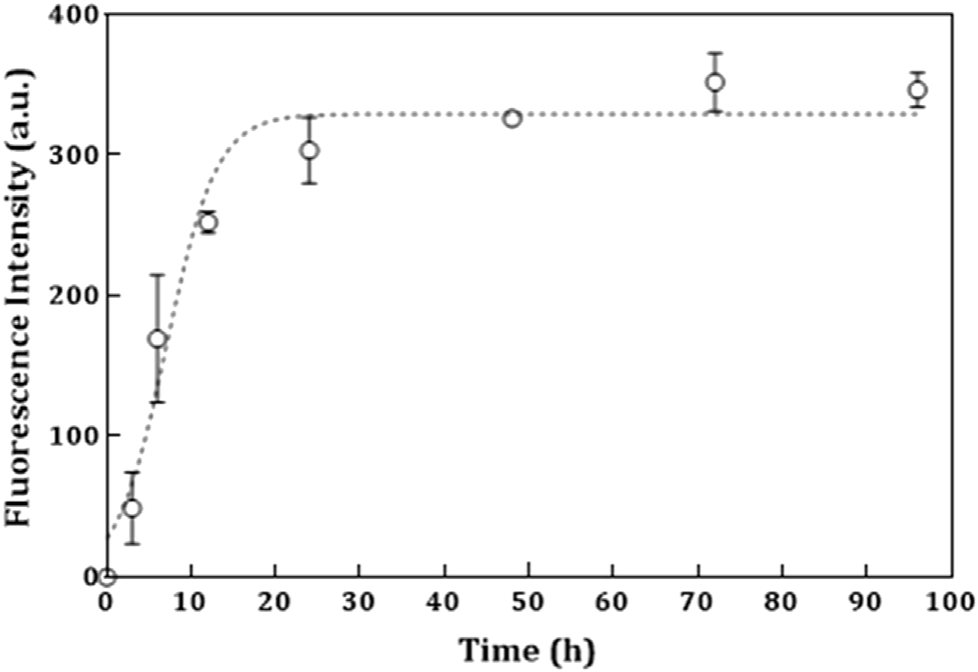
Kinetics studies of tau protein fibril formation induced by heparin in the presence of DTT. ThT fluorescence used to monitor kinetics of 20 µM tau protein induced by 5 M heparin in the presence of DTT at 37°C in 50 mM Tris-HCl buffer of pH 7.6 and 100 mM NaCl. The continuous line through the data points is a least square fit to a sigmoidal equation. The error bars represent the spread in the data calculated from three independent experiments. The kinetic parameter k_app_ (the apparent rate constant) was calculated to be 2.78 × 10^–1^ h^−1^, with Tm of 7.498 h and Fmax of 346.6.

Analysis of nucleation and elongation steps involved in the tau protein fibril formation may be useful for gaining a deeper insight into possible mechanisms of amyloid formation and would identify valuable therapeutic targets for the treatment of related neurodegenerative diseases (Chiti and Dobson, 2006). Heparin induces the formation of tau filaments *in vitro* with very similar morphologies to that *in vivo* (Ramachandran and Udgaonkar, 2011). Therefore, heparin-induced polymerization of 1N4R tau protein was achieved *in vitro* and analyzed by the ThT-binding assay as most commonly used to assess the amyloid assembly in real time. ThT shows enhanced fluorescence at 485 nm when bound to amyloid fibrils (Sahara et al., 2007). Evidence reveals that the intermolecular tau cross-linking of disulfides is critical in the production of tau oligomers, which act as a building block for higher-order aggregates (Haque et al., 2014). Therefore, providing reductive conditions is equivalent to providing tau in the form of free monomers in the early stages, as well as allowing the cysteine residues to be involved in the formation of oligomers. To investigate the mechanism of tau fibril formation, the time course of tau fibril formation induced by heparin was measured in the presence of DTT. Tau protein induced by heparin, generally, displayed an increase in the fluorescence signal only after 4–5 h of incubation. As shown in Figure 2, the fibril formation process was found to obey the characteristic nucleation–elongation pattern, with three distinct phases: initial nucleation (assembly of monomers), elongation (formation of oligomers and subsequent fibrils), and equilibration (saturation) (Teplow, 1998; Ramachandran and Udgaonkar, 2011). It should be added here that, in the sequence of the 4R isoform of tau, there are two cysteines that can be involved in forming disulfide bonds, via intramolecular or intermolecular interactions (Sahara et al., 2007). Therefore, reducing conditions play an important role in directing aggregates to amyloid fibrils. The tau isoform used in this study was able to undergo a corresponding structural change to form non-covalent dimers (Mostafavi et al., 2017). This may explain why the presence of DTT in addition to heparin is necessary in the fibrillation process of tau.

#### 3.1.3 SDS-PAGE Analysis of Tau Protein Monomers and Formation of Oligomeric Species Over Time

**FIGURE 3:**
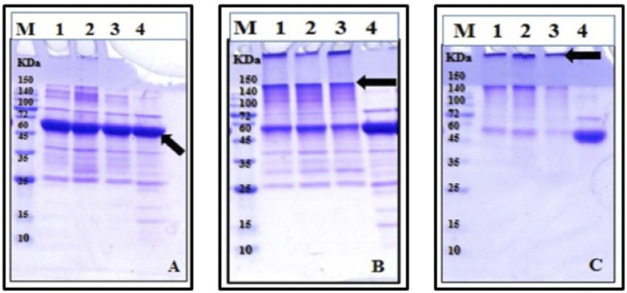
SDS-PAGE analysis of tau protein fibril formation. Tau protein was incubated for different periods of time: **(A)** 6 h, **(B)** 48 h, and **(C)** 96 h, with 5 mM heparin in the presence of DTT and then analyzed by 12% SDS polyacrylamide gels, followed by protein staining with Coomassie Brilliant Blue. The sample description in gels **(A–C)** is the same except for different incubation periods. M, protein marker; lanes 1–3, three replicates of the not-treated sample in the presence of DTT; lane 4, not-heated control tau protein in the presence of DTT.

To obtain information on the fibrillation kinetics of tau protein aggregation intermediates, the samples from the fibrillation experiment were examined after different periods of time and subjected to SDS-PAGE analysis. Upon analysis, it was apparent that the monomeric native tau protein ran as a single band with an approximate molecular weight (MW) below the 60 kDa marker band (Figure 3A, lane 4, arrow). At various increasing time intervals of tau incubation (under fibrillation conditions), additional bands appeared in the range of the ∼100–150 kDa protein marker bands and increased in MW (Figure 3B, lanes 1–3, arrow). Therefore, over time, a reduction in the amount of monomers was seen leading to an increase in the aggregated forms of tau protein, including dimers, oligomers, and fibrils. After 48 h (Figure 3B, lanes 1–3), this pattern became quite visible, so that gradually after further incubation at 96 h, the larger aggregated/fibrillar forms of tau protein did not enter the gel, and after staining, the bands were observed in the wells of the gel (Figure 3C, lanes 1–3, arrow). It has been reported that the higher MW tau protein filaments, which do not enter the stacking gel, could not be quantified via SDS-PAGE (Flach et al., 2012). Therefore, the wells of the SDS polyacrylamide gels in this study were assessed to detect the protein fractions, which did not enter the gels. The SDS-PAGE results also confirmed that the distinct higher MW bands located above the monomeric tau protein band were mainly seen after prolonged incubation (Figures 3B,C, lanes 1–3), while the not-heated tau protein, as a control on each gel (Figures 3A–C, lane 4), did not show the formation of these higher MW species of tau protein. This was previously reported to be the case (Perez et al., 2001; Goux et al., 2004).

#### 3.1.4 ThT Results for Tau Protein After 96 h of Incubation

**FIGURE 4:**
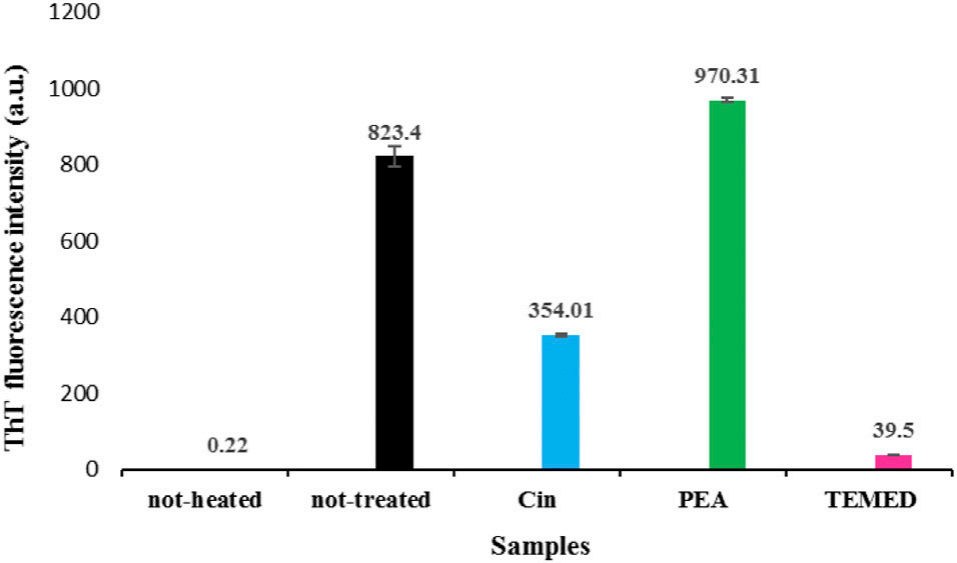
ThT analysis of tau fibrillation in the presence and absence of the volatile compounds Cin, PEA, and TEMED, after 96 h of incubation. The results are based on an average of two to three repetitions, and the difference in the intensities of the emissions are compared to that of the two controls including the not-heated and not-treated samples. Tau protein samples were used in fibrillation studies as described by the setup in Figure 1. ThT emission was measured at 484 nm.

The ThT-binding assay was obtained for tau samples in the presence and absence of each of the volatile compounds Cin, PEA, and TEMED, after 96 h incubation under fibrillation conditions. As revealed in Figure 4, ThT intensity was increased for the not-treated sample, which confirmed formation of the fibrillar form of tau. The presence of Cin and in particular TEMED resulted in a reduction in the intensity of ThT fluorescence compared to that of the not-treated sample. However, the results were non-conclusive, especially since there was a great increase in the ThT intensity for the tau protein sample incubated in the presence of PEA. Since it has been previously mentioned that ThT data need to be supported by other techniques (Seraj et al., 2018), the ThT data alone did not provide the necessary data for a conclusion to be made. Therefore, other techniques were used to arrive at a better conclusion.

#### 3.1.5 Effects of the Volatile Compounds Cin, PEA, and TEMED on Tau Fibrillation as Assessed by SDS-PAGE

**FIGURE 5:**
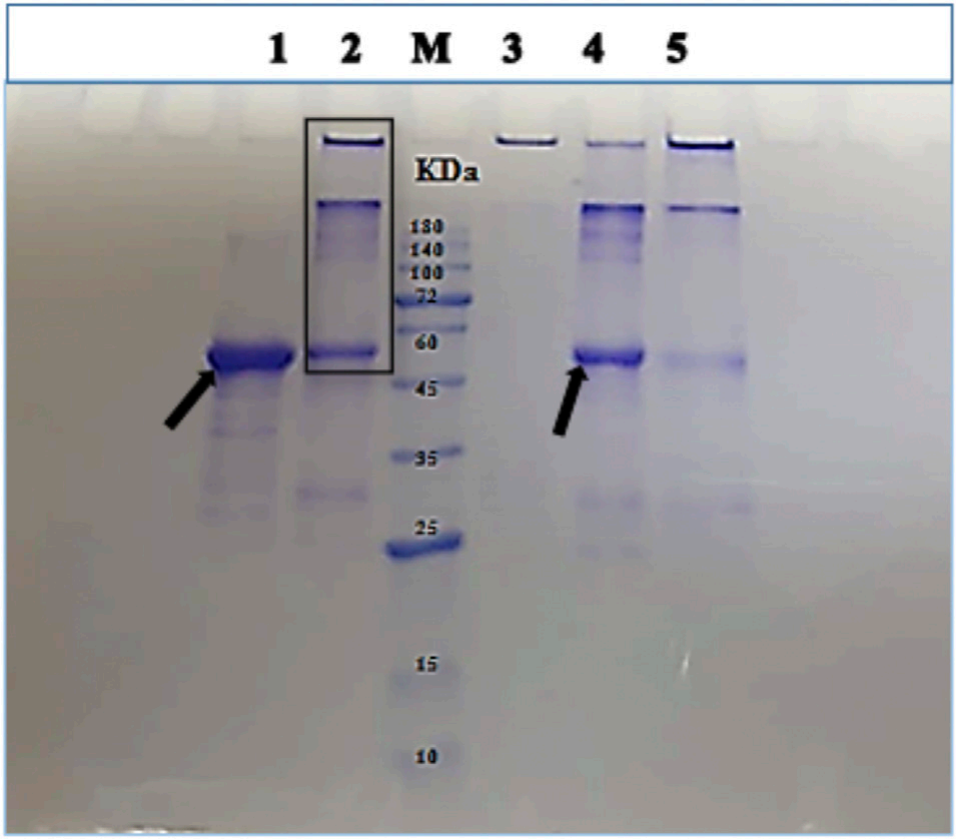
SDS-PAGE analysis of tau protein in the presence and absence of the volatile compounds Cin, PEA, and TEMED, after 96 h of incubation under fibrillation conditions. Gel lanes are as follows: 1 and 2, not-heated and not-treated tau protein controls, respectively; M, protein marker; 3–5, tau protein treated with Cin, PEA, and TEMED, respectively. Tau protein samples were used in fibrillation studies as described by the setup in Figure 1.

The effect of the volatile compounds Cin, PEA, and TEMED on tau fibrillation was assessed via SDS-PAGE (Figure 5). It was observed that the not-heated monomeric tau protein migrated into the gel below the 60 kDa protein marker band (Figure 5, lane 1; shown by an arrow). This revealed that the not-heated sample was able to enter the 12% gel. However, a large portion of the not-treated tau protein was found in the well, and much less protein was detected at the same position where the monomeric tau protein would be seen (Figure 5; lane 2). It has been reported that, during the aggregation stages of recombinant tau *in vitro*, dimers with apparent sizes of 180 kDa (Patterson et al., 2011) and 130 kDa (Makrides et al., 2003) as well as trimers with an apparent size of 120 kDa (Lasagna-Reeves et al., 2010) are observed. As for tau treated with Cin, no protein band was seen migrating into the gel (Figure 5, lane 3). This may indicate that oligomeric forms of tau protein were produced because oligomers are considered too large to enter the gel compared to protofibrils (Ross and Poirier, 2004). As for tau protein treated with PEA, the intensity of the main monomeric tau protein band increased in the gel (Figure 5, lane 4, shown by an arrow) compared to that of the not-treated tau protein sample, indicating that perhaps PEA was able to inhibit mature fibril formation to some extent. Additionally, there was an obvious reduction in band intensity of fibrillar form of tau protein in the well of lane 4. Treatment of tau protein with TEMED, on the contrary, resulted in a large increase in the amount of fibrillar tau protein in the well (lane 5), with only a low intensity band observed for the tau monomeric protein, migrating into the gel. It is plausible to suggest that when tau protein was treated with TEMED, insoluble mature fibrils were formed, which could not enter the gel.

#### 3.1.6 Quantification of Tau Protein Band Intensities in SDS-PAGE as Analyzed by ImageJ Software

**FIGURE 6:**
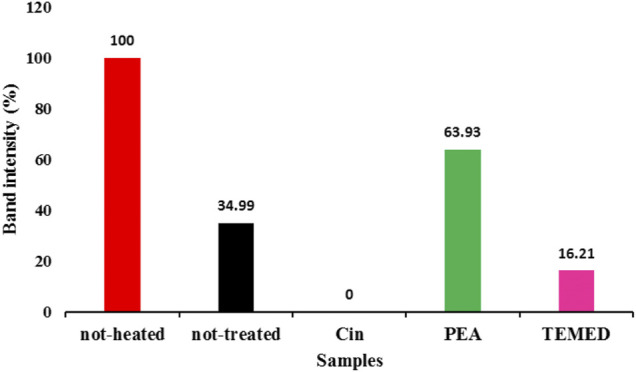
Quantitative measurement of the monomeric form of tau protein band by ImageJ software. The quantification of the monomeric form of tau protein band is related to the SDS-PAGE samples described in Figure 5.

The ImageJ software is used for processing and quantifying images (Hartig, 2013). One of its applications is to convert the intensity of electrophoresis gel bands to quantitative data. Figure 6 shows the result of this quantification by considering the monomeric band intensity of the tau protein samples entering the gel, with a base value of 100% set for the not-heated sample, as analyzed by SDS-PAGE in Section 3.1.5. Compared to the not-treated tau protein with 34.99% band intensity for the monomeric protein entering the gel, quantification data showed that, in the presence of PEA, the amount of monomeric form of tau protein entering the gel increased to 63.93%, indicating a reduction in the amount of aggregated tau protein upon PEA treatment. In contrast, TEMED seemed to increase the aggregation of tau protein, since it only allowed 16.21% of the monomeric form of tau protein to enter the gel. In the presence of Cin, no protein band was seen entering the gel.

Therefore, the SDS-PAGE analysis and the quantification of the monomeric band for tau protein observed in the gel were indicative of the positive effect of PEA in preventing tau protein aggregation.

#### 3.1.7 Secondary Structure Changes of Tau Protein as Analyzed by CD

**FIGURE 7:**
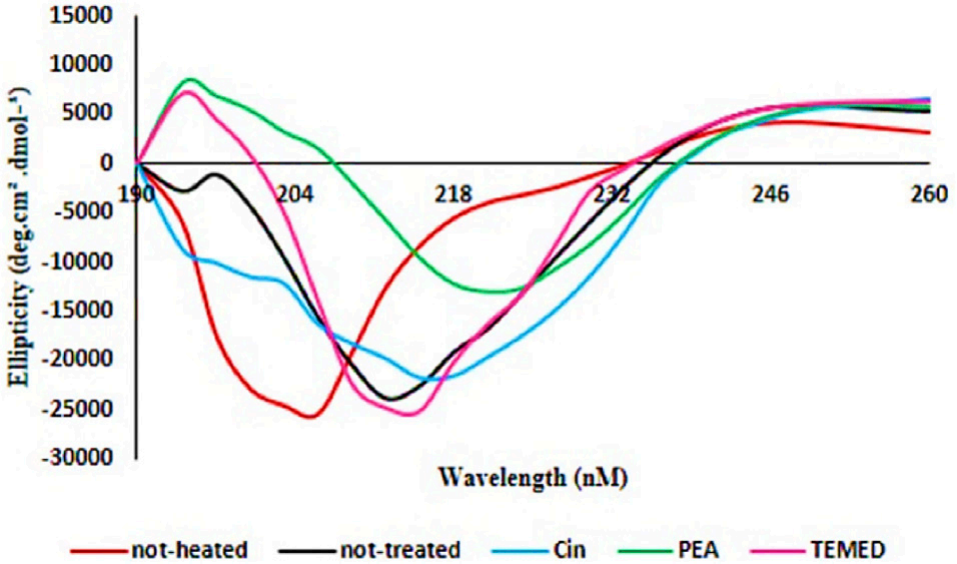
Far-UV CD spectra of tau protein incubated with and without the volatile compounds Cin, PEA, and TEMED. The CD spectra of not-heated tau protein compared to tau protein incubated in the presence or absence of volatile compounds Cin, PEA and TEMED are shown.

Far-UV CD spectroscopy was used to gain an insight into the secondary structure change of tau protein, under the effect of the volatile compounds (Figure 7). CD data revealed that the soluble native tau protein (not-heated) exhibited a predominantly random coil structure in agreement with previously reported data (von Bergen et al., 2005; Friedhoff et al., 2000). After 96 h of tau protein being induced by heparin in the presence of DTT, a transition away from random coil was detected. The spectra of the not-treated tau protein aggregation revealed a clear shift from lower to higher wavelengths and a broad minimum at around 217 nm, as a typical feature of the beta-sheet structure (Chirita et al., 2005). The sample of tau protein under the influence of PEA was associated with a decrease in the intensity of ellipticity, with a broad minimum at around 220 nm. It is reported that the ellipticity degree decreased at ∼ 220 nm could be related to the conversion of toxic amyloid structures to non-toxic beta-sheet–rich fibers in the presence of specific molecules (Bieschke et al., 2012). Cin was also able to reduce the ellipticity degree in the negative region, but much less than PEA. TEMED, on the contrary, was seen with a negative peak at about 217 nm, similar to the not-treated tau protein spectrum. These results demonstrated that the volatile compounds had an effect on the structure and aggregation tendency of the tau protein.

#### 3.1.8 Characterization of Tau Protein Fibril Morphology

**FIGURE 8:**
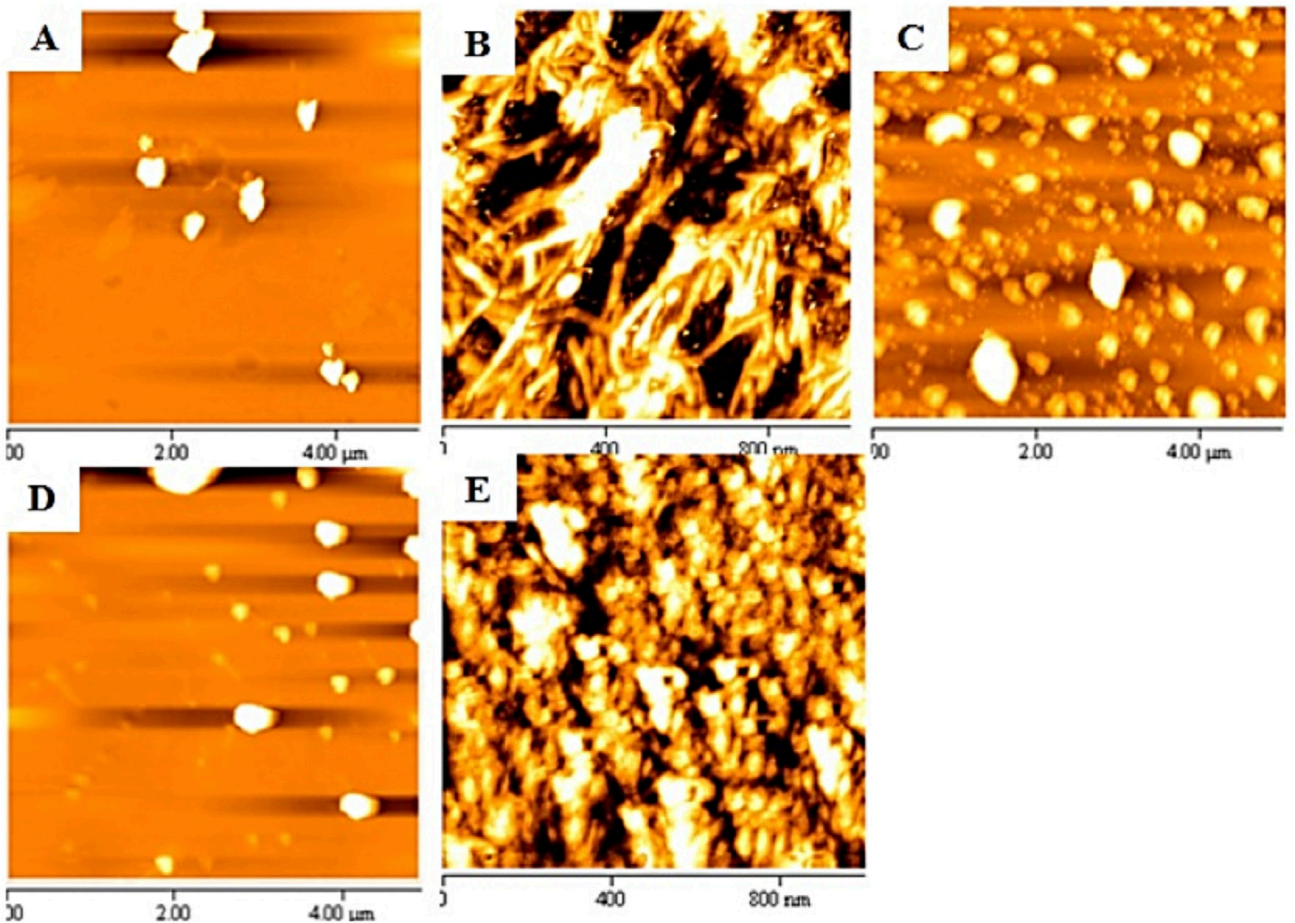
AFM images of samples of tau protein incubated in the presence or absence of the volatile compounds Cin, PEA, and TEMED. The images show **(A)** not-heated tau protein, **(B)** not-treated tau protein, **(C)** tau protein treated with Cin, **(D)** tau protein treated with PEA, and **(E)** tau protein treated with TEMED.

Atomic force microscopy (AFM) was used to investigate the structure and morphology of the incubated samples in the presence or absence of the volatile compounds Cin, PEA, and TEMED. Figure 8 shows the morphology of tau protein aggregates in the presence and absence of the three different volatile compounds. In the not-heated tau protein, no aggregation was observed (Figure 8A), while incubation of tau protein in the presence of heparin and DTT for 96 h at 37°C resulted in protein aggregation, where fibrillary aggregates were of the dominant form (Figure 8B). Based on the AFM results, Cin seemed to have led to the formation of granular-shaped oligomers in tau protein. In some areas, these oligomers were seen to be close to each other (Figure 8C). However, previous studies have shown that some aggregates of tau, especially oligomeric forms, are non-toxic and may even play a protective role in biological conditions against the toxicity of tau protein itself (Cowan and Mudher, 2013). PEA, on the contrary, appeared to largely inhibit the aggregation process (Figure 8D), and the AFM morphology looked very similar to that of the not-heated sample. The difference in the morphology of the aggregates under the influence of TEMED (Figure 8E) was quite obvious, when compared to the AFM image for not-treated tau protein; it appeared to be of the mature fibrillar form of tau protein, even more condense than that of the not-treated sample.

### 3.2 α-Synuclein Protein Studies

#### 3.2.1 α-Synuclein Protein Over-Production and Purification

The pT7-7 WT α-synuclein protein which encoded the 140 amino acids of the protein was a gift from Professor Jun Hong (School of Life Sciences, Henan University, Kaifeng, China). The untagged recombinant WT α-synuclein protein was over-produced in BL21 (DE3) cells. The α-synuclein protein was purified using boiling, ammonium sulfate precipitation, and HiTrap Q-Sepharose FF anion exchange chromatography. The over-produced and purified protein was assessed using 12% Bis-Tris gel as revealed in Supplementary Figure S2. Fractions containing protein corresponding to the predicted monomeric α-synuclein molecular weight (MW) of 14.4 kDa were used and dialyzed against PBS, pH 7.4. Protein concentration was determined by absorbance at 275 nm, using an extinction coefficient of 5600 M^−1^cm^−1^.

#### 3.2.2 ThT Results for α-Synuclein After 3 Days of Incubation

**FIGURE 9:**
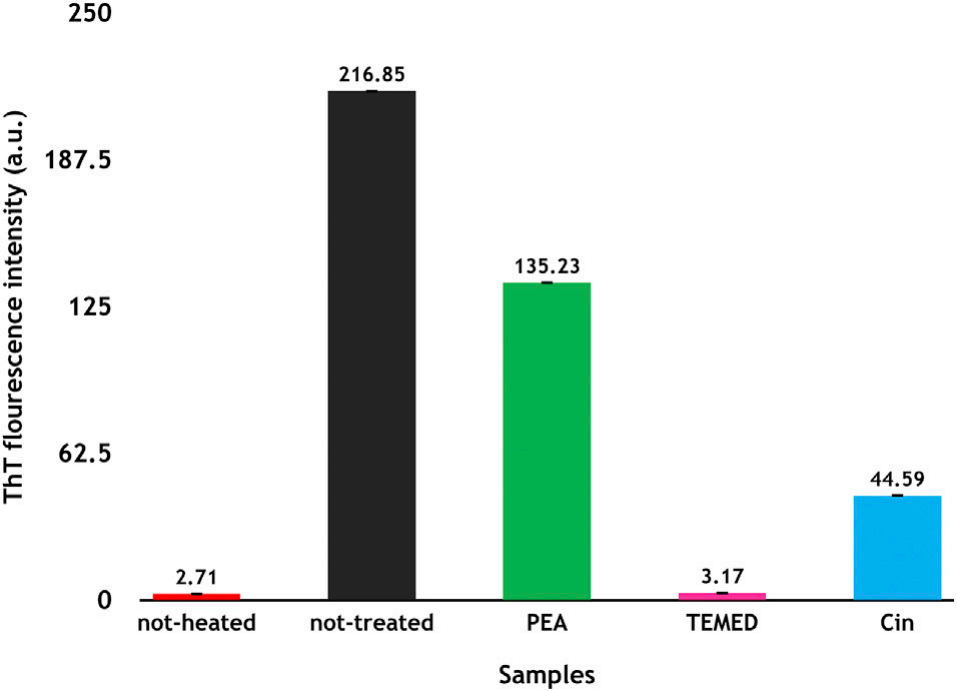
ThT analyses of α-synuclein fibrillation in the presence and absence of the volatile compounds Cin, PEA, and TEMED after 3 days of incubation. The results are based on an average of two to three repetitions, and the difference in the intensities of the emissions is compared to that of the two controls including the not-heated and not-treated samples. ThT emission was measured at 484 nm.

The ThT-binding assay was obtained for α-synuclein samples in the presence and absence of each of the volatile compounds after 72 h incubation of protein under fibrillation conditions. As revealed in Figure 9, ThT intensity was increased for the not-treated sample, indicating formation of fibrillar form of α-synuclein. Although the presence of Cin and TEMED, in particular, resulted in the reduction of ThT intensity compared to that of the not-treated sample, the results were non-conclusive as ThT data needed to be accompanied by other techniques to be understood better (Seraj et al., 2018).

#### 3.2.3 Effects of the Volatile Compounds Cin, PEA, and TEMED on α-Synuclein Fibrillation as Assessed by SDS-PAGE and Native-PAGE

**FIGURE 10:**
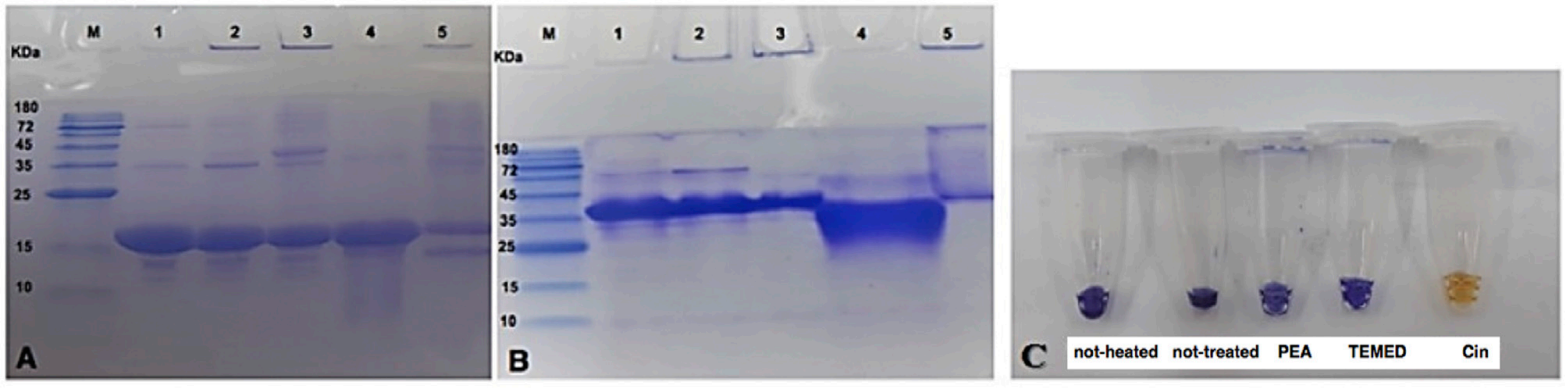
SDS- and native-PAGE analysis of not-heated, not-treated, and treated α-synuclein samples with the volatile compounds Cin, PEA, and TEMED after 72 h of incubation. **(A)** SDS-PAGE results. **(B)** Native-PAGE results. Gel lanes are as follows: M, marker; 1, not-heated α-synuclein; 2, not-treated α-synuclein; 3, α-synuclein treated with PEA; 4, α-synuclein treated with TEMED; 5, α-synuclein treated with Cin. **(C)** Color observation of SDS-PAGE loading buffer upon addition of not-heated and not-treated α-synuclein control samples and that of samples of α-synuclein treated with PEA, TEMED and Cin. Only in the sample of α-synuclein treated with Cin, the loading buffer color changes from blue to orange.

To evaluate the effect of the volatile compounds Cin, PEA, and TEMED on α-synuclein under fibrillation conditions after 72 h, the samples were analyzed by SDS- and native-PAGE. As shown in Figure 10A, lane 1, the not-heated monomeric α-synuclein protein sample, assessed by SDS-PAGE, was seen to be close to the 15 kDa marker band. However, α-synuclein from the same sample, as assessed by native-PAGE, was seen to be larger than the 35 kDa marker band (Figure 10B, lane 1). This result is consistent with a previous report about the structure of α-synuclein (Li et al., 2018), which showed the presence of the dimerized protein due to the formation of a steric zipper core composed of residues 50–57 (HGVATVAE), via hydrophobic and electrostatic (non-covalent) interactions. Additionally, appearance of some extra protein bands near the 35 and 72 kDa marker bands as analyzed by SDS-PAGE could be representative of oligomers of the protein, even in the not-heated protein. The presence of these bands in lanes 2, 3, and 5, and their relative absence in lane 4 of Figure 10A, confirmed that they are not artifacts or impurities. When α-synuclein was incubated for 72 h under fibrillation conditions and in the absence of any treatment, the extra band at around the 35 kDa marker band on the SDS gel and around the 72 kDa marker band on the native gel became more obvious (Figures 10A,B, lane 2). Furthermore, the presence of Coomassie stain in the wells of lanes 2, 3, and 5 of Figure 10A suggested formation of insoluble oligomers/fibrils that were not able to enter the gels. Looking at the treated samples (Figures 10A,B, lanes 3–5), although in the presence of PEA there was not a considerable change in the intensity of Coomassie stain in the well of the gel (lane 3), compared to the intensity of Coomassie stain in the well of the not-treated sample (lane 2), there was no Coomassie stain in the well of the sample of α-synuclein treated with TEMED (lane 4), with almost complete elimination of possible oligomeric forms of the protein entering the gel, which could otherwise be seen in the sample of not-treated α-synuclein and to a lesser extent in the sample of α-synuclein treated with PEA. As for α-synuclein treated with Cin, a lower intensity Coomassie stain was seen in the well of the gel, as well as lower intensity bands for possible oligomeric forms of the protein. However, some kind of lower MW degradation product of α-synuclein was observed (Figure 10A, lane 5). It is necessary to add here that Cin can convert to cinnamic acid in solution and that it has been reported that cinnamic acid derivatives can prevent amyloid transformation of α-synuclein, also resulting in susceptibility of α-synuclein to proteolytic cleavage (Medvedeva et al., 2020). On this note, it should be added that there was a transient observation of color change from blue to orange of the SDS-PAGE loading buffer for the sample of α-synuclein treated with Cin (Figure 10C). This kind of color change was previously seen when a sample with acidic pH was added to the SDS-PAGE loading buffer (Seraj et al., 2018). The observation of the transient color change could explain the presence of cinnamic acid in the sample of α-synuclein treated with Cin and furthermore be a possible reason for the detection of the degradation product.

#### 3.2.4 Characterization of α-Synuclein Protein Fibril Morphology

**FIGURE 11:**
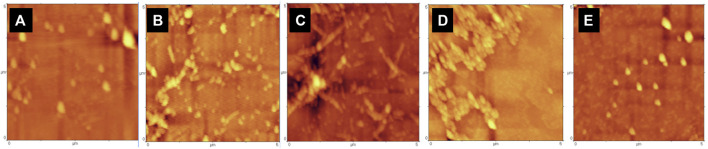
AFM result of α-synuclein control samples and samples treated with the volatile compounds Cin, PEA, and TEMED after 72 h of incubation. **(A)** Not-heated α-synuclein; **(B)** not-treated α-synuclein; **(C)** α-synuclein treated with PEA; **(D)** α-synuclein treated with TEMED; **(E)** α-synuclein treated with Cin.

To further our understanding about the effects of Cin, PEA, and TEMED on the fibrillation process of α-synuclein, AFM was used to analyze the protein fibril morphology. The AFM morphologies related to not-heated and not-treated α-synuclein samples showed confirming results as controls, where there was no fibrillation seen in the not-heated sample, whereas early fibrils were observed for the not-treated α-synuclein sample (Figures 11A,B). It is speculated that the fibrillation process was not complete and that the duration of 72 h was not enough for the mature fibrils to form. This is supported by the low value ThT intensity for the not-treated sample and the relatively weak band intensity of fibrillar form of α-synuclein in the wells of the SDS and native gels. Nevertheless, the AFM data obtained for the α-synuclein sample treated with PEA suggested a protofibrillar form of the protein (Figure 11C). This could explain why the ThT intensity was high for this sample. The effect of TEMED, however, was suggestive of amorphous aggregates, and not fibrils (Figure 11D), and that could be why the ThT intensity was so low for this sample. As for the AFM result for α-synuclein treated with Cin, due to the acidic pH and the probable degradation of the protein, there were no signs of fibrillation/aggregation (Figure 11E). Oligomers may have formed, however, which did not enter the gel, as revealed by SDS- and native-PAGE results (Figures 10A,B).

#### 3.2.5 Effects of the Volatile Compounds Cin, PEA, and TEMED on α-Synuclein Fibrillation as Assessed by DLS

**FIGURE 12:**
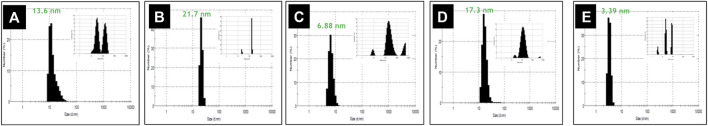
DLS result of α-synuclein control samples and samples treated with volatile compounds Cin, PEA, and TEMED after 72 h of incubation. **(A)** Not-heated α-synuclein; **(B)** not-treated α-synuclein; **(C)** α-synuclein treated with PEA; **(D)** α-synuclein treated with TEMED; **(E)** α-synuclein treated with Cin. The DLS results in the intensity mode for each sample are given as inset to panels **(A–E)**.

To better understand the results obtained for the effects of the volatile compounds Cin, PEA, and TEMED on α-synuclein fibrillation, dynamic light scattering (DLS) was used. As shown in Figure 12, although the change in diameter size was not great, the not-treated sample of α-synuclein had a diameter of 21.7 nm (Figure 12B), larger than that of the not-heated α-synuclein sample at 13.8 nm (Figure 12A). As for α-synuclein treated with PEA, the diameter size had been reduced to 6.88 nm (Figure 12C), while contrary to the ThT and SDS- and native-PAGE results, the diameter size for α-synuclein treated with TEMED was larger at 17.3 nm (Figure 12D), indicative of larger aggregated forms of α-synuclein. For the α-synuclein sample treated with Cin, the diameter size had been reduced significantly to 3.39 nm (Figure 12E), which could be explained by the acidic pH observed and the probable degradation product produced.

## 4 Conclusion

Among several therapeutic approaches used against Alzheimer’s and Parkinson’s diseases in recent years, efforts have been made to use interfering molecules as fibrillation or aggregation inhibitors. The present study was designed to investigate the molecular effects of the volatile compounds Cin, PEA, and TEMED on disease-related tau and α-synuclein fibrillation processes. In our previous studies, HEWL was used as a model protein for fibrillation studies (Seraj et al., 2018), where it was concluded that HEWL was not the correct protein to be used as a model protein since TEMED, a diamine with bad odour/smell, similar to putrescine and cadaverine, known as the “smell of death” molecules, was able to completely prevent fibrillation of HEWL. This was explained to be due to the fact that putrescine and cadaverine, as by-products of amino acid degradation through bacterial infection, provide the foul smell, which can then act as an activation signal for lysozyme to help clear up the infection site (Seraj et al., 2020). Based on these findings and the exclusion of HEWL as the correct model protein, as well as the findings that Cin and PEA promoted the entrapment of intermediate species of HEWL (through structural, kinetics, and thermal stability studies) as a globular protein (Seraj et al., 2019), different in structure from tau and α-synuclein proteins, which are unstructured proteins, it was important to analyze the effect of the three volatiles compounds on the correct protein, directly related to the brain and the related neurodegenerative diseases concerned.

As such, the findings from this study enabled us to make a good comparison between the data previously obtained on HEWL and those of tau and α-synuclein proteins.

Looking at the effect of PEA on tau protein, the results from SDS-PAGE, CD, and AFM collectively provided evidence that PEA had the potential to prevent fibril formation; a good amount of monomeric form of tau protein entered the SDS polyacrylamide gel, Far-UV CD results revealed a decrease in the intensity of ellipticity, with a broad minimum at around 220 nm, which could be related to the conversion of toxic amyloid structures to non-toxic beta-sheet–rich fibers (Bieschke et al., 2012), and AFM revealed a morphology similar to the not-heated tau sample. As for the effect of PEA on α-synuclein, although the ThT intensity was high (but lower than that of the not-treated sample), the DLS diameter size was lower than that of the not-treated and not-heated proteins, and a different type of morphology to fibrils was seen in AFM results, suggesting that possibly the effect of PEA resulted in a mixture of native, oligomeric, and protofibrillar forms of α-synuclein. However, in comparison, when HEWL was treated with PEA-L (Seraj et al., 2018), while the ThT intensity was high (lower than that of the not-treated sample), the DLS diameter size was much larger than that of the not-heated HEWL, and oligomeric forms of HEWL could be observed in AFM results. Additionally, the majority of the HEWL protein did not enter the SDS gel and was observed in the well. This was not true for α-synuclein, indicating that perhaps the effect of PEA on α-synuclein allowed retainment of the native form to a considerable level, and as for tau, PEA was able to prevent fibril formation to a considerable level.

As for the effect of Cin, when tau protein was treated with Cin, an oligomeric form of tau was seen, as evidenced by the inability of tau protein to enter the SDS gel, the shift in the far-UV CD spectrum (somewhat similar to that seen in PEA), and the non-fibrillar morphology in the AFM image. When α-synuclein was treated with Cin, there were a great reduction in the ThT intensity (compared to that of the not-treated sample) and possible degradation of the protein, where an additional lower MW band was seen on the SDS polyacrylamide gel. Furthermore, DLS results revealed that Cin resulted in a reduced diameter size (even smaller than that of the not-heated sample), and AFM results further showed that there were no signs of aggregates/fibrils. These observations can be explained by the conversion of Cin to cinnamic acid in solution (supporting the transient color change in the SDS-PAGE loading buffer from blue to orange, indicative of an acidic pH). Overall, as explained previously, cinnamic acid derivatives have been reported to prevent amyloid transformation of α-synuclein, also resulting in susceptibility of α-synuclein to proteolytic cleavage (Medvedeva et al., 2020). Therefore, our results reveal that Cin may have prevented fibril formation of α-synuclein.

For comparison with previous data using HEWL, the most revealing and significant result comes from the effect of TEMED. In this study, tau treated with TEMED, although having the lowest ThT intensity, revealed the lowest amount of monomeric protein entering the SDS polyacrylamide gel and showed beta sheet content as revealed by far-UV CD analysis and fibrillar morphology as revealed by AFM. As for α-synuclein, although treatment with TEMED also revealed a low ThT intensity, AFM showed amorphous aggregates being formed, and the diameter size detected by DLS was shown to be quite large. In contrast, HEWL treated with TEMED-L had revealed low ThT intensity and smaller than native diameter size with AFM, revealing a non-fibrillar form of the protein similar to the not-heated sample AFM morphology. Therefore, it is plausible to conclude that the effect of TEMED was significantly different between the globular HEWL structure and the unstructured tau and α-synuclein proteins. Therefore, when comparing the effect of TEMED on tau and α-synuclein and that of TEMED-L on HEWL (Seraj et al., 2018), it can be said that while TEMED in this study led to the formation of mature fibrils of tau and amorphous aggregates of α-synuclein, respectively, TEMED-L was able to completely stop the fibrillation or aggregation of HEWL and maintain the native structure and activity of HEWL, as revealed by activity results of the enzyme (Seraj et al., 2018; Seraj et al., 2020).

Finally, the data from this study have shown that PEA had the ability to prevent the formation of fibrils to a good extent, particularly in tau protein. Cin, on the contrary, seemed to prevent the formation of mature fibrils by creating oligomeric forms of tau as intermediate species in the fibrillation pathway. This type of oligomeric form may be the non-toxic type as have been mentioned to exist (Cowan and Mudher, 2013). TEMED, however, as a representative of the smell of death, showed an increase in the process of tau aggregation and resulted in amorphous aggregates in α-synuclein. Overall, it is suggested that the volatile compounds PEA and Cin should be further studied as they may be potential therapeutic candidates for prevention of tau or α-synuclein fibrillation related to AD/PD, respectively, as well as other diseases such as SARS-CoV-2, where stimulating the sense of smell can improve patients’ cognitive function and allow nerve regrowth through odour-producing compounds.

## Abbreviations

AD, Alzheimer’s disease; AFM, atomic force microscopy; Cin, cinnamaldehyde; CD, circular dichroism; DLS, dynamic light scattering; PEA, phenyl ethyl alcohol; TEMED, N,N,N′,N′-tetramethylethylenediamine; HEWL, hen egg white lysozyme; PD, Parkinson’s disease; ThT, thioflavin T.

## Data Availability

The original contributions presented in the study are included in the article/Supplementary Material, further inquiries can be directed to the corresponding author.
