# Supplementary material for: Unravelling the Novel Effects of Three Volatile Compounds in Preventing Fibril Formation of Disease Related Tau and α-Synuclein Proteins- Towards Identifying Candidate Aromatic Substances for Treating Neurodegenerative Diseases
Source: Front Pharmacol. 2022 Mar 22;13:793727. doi: 10.3389/fphar.2022.793727 (PMC8980687; doi:10.3389/fphar.2022.793727)
Supplement: Supplementary file 1 [file DataSheet1.pdf]

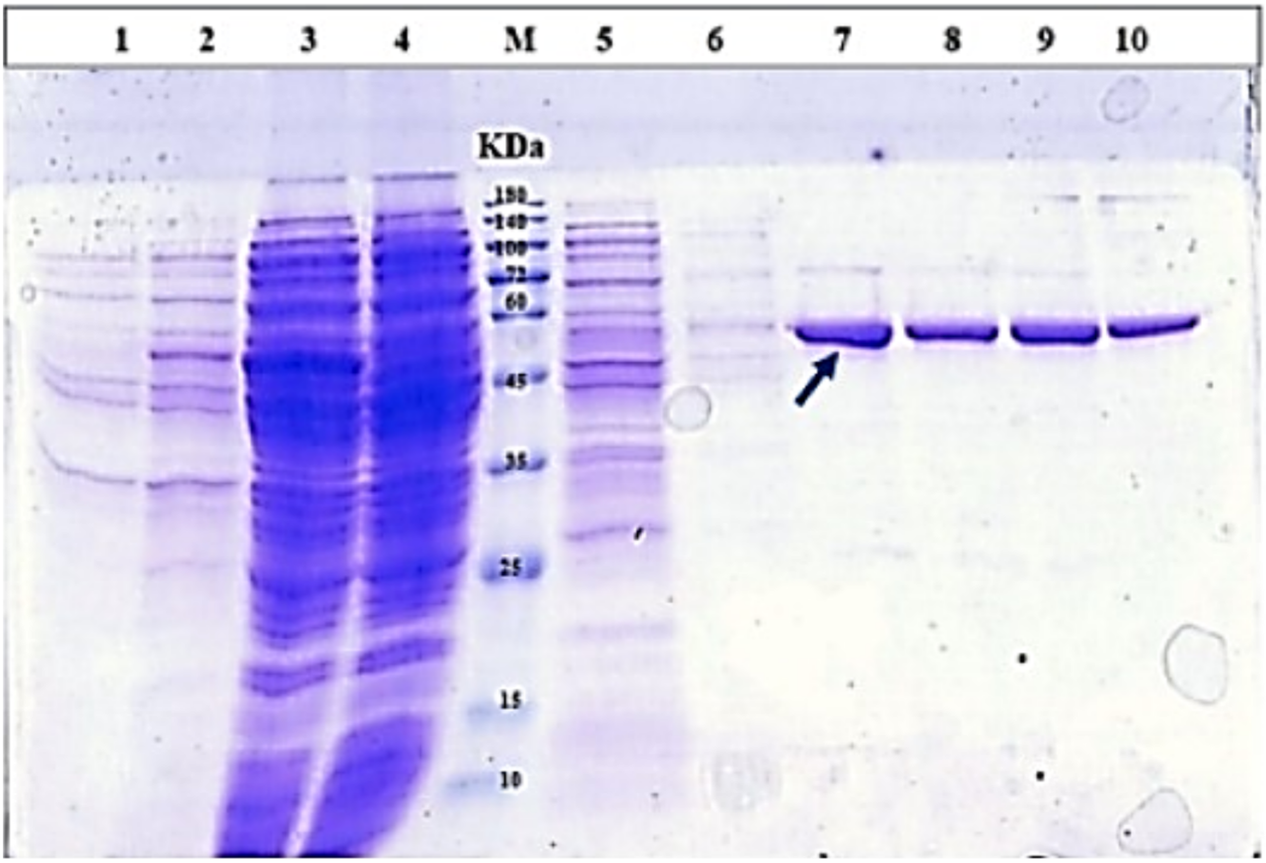

Figure S1. Over-production and purification of the human 412 residue tau protein. The samples were mixed with SDS-sample buffer, separated on 12 % SDS-polyacrylamide gel and stained with Coomassie Brilliant Blue R250. The tau protein band is close to the 60 kDa marker band, shown by an arrow. Lane 1: sample before IPTG-induction, 2: sample after IPTG-induction, 3: post-sonicated supernatant, 4: flow-through from the nickel chelating sepharose beads, M: protein Marker, 5 and 6: wash samples from the beads and 7-10: eluted tau protein samples after purification using Ni−NTA−Agarose precipitation.

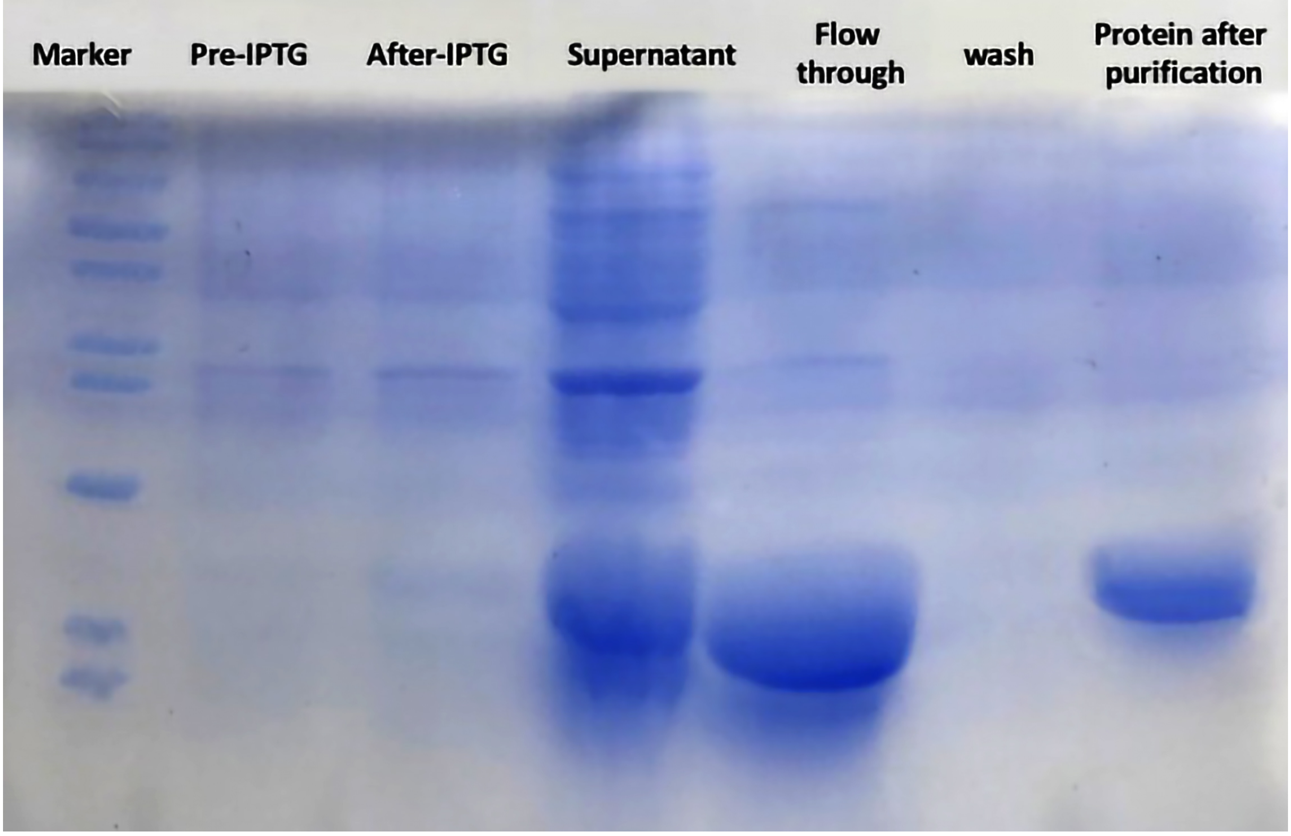

Figure S2. Over-production and purification of recombinant human α-synuclein. Lane 1: molecular marker; lane 2: sample before IPTG-induction; lane 3: sample after IPTG-induction; lane 4: supernatant after sonication; lane 5: flow-through; lane 6: wash sample; lane 7: protein after purification on a Q-Sepharose Fast Flow column. The samples were mixed with SDS-sample buffer, separated on 18 % SDS-polyacrylamide gel and stained with Coomassie Brilliant Blue R250. The α-synuclein band is close to the ~14 kDa marker band.
